# Can Immersive Virtual Reality Videogames Help Parkinson’s Disease Patients? A Case Study

**DOI:** 10.3390/s21144825

**Published:** 2021-07-15

**Authors:** Pablo Campo-Prieto, Gustavo Rodríguez-Fuentes, José Mª Cancela-Carral

**Affiliations:** 1 HealthyFit Research Group, Department of Functional Biology and Health Sciences, Faculty of Physiotherapy, Galicia Sur Health Research Institute (IIS Galicia Sur), University of Vigo, E-36005 Pontevedra, Spain; pcampo@uvigo.es (P.C.-P.); gfuentes@uvigo.es (G.R.-F.); 2 HealthyFit Research Group, Department of Special Didactics, Faculty of Education and Sports Science, Galicia Sur Health Research Institute (IIS Galicia Sur), University of Vigo, E-36005 Pontevedra, Spain

**Keywords:** virtual reality exposure therapy, Parkinson’s disease, older adults, video games, exergaming, neurological rehabilitation, games for health

## Abstract

Video games have proven useful in physical rehabilitation therapy. Accessibility, however, is limited for some groups such as the elderly or patients with Parkinson’s disease (PD). We explore the potential of fully immersive video games as a rehabilitation tool in PD patients. Four patients with mild-moderate PD (3 males:1 female, 53–71 years) participated in the study. Training consisted in two immersive virtual reality video gaming sessions. Outcomes were evaluated using System Usability Scale (SUS), Simulator Sickness Questionnaire (SSQ), Game Experience Questionnaire-post game (GEQ), an ad hoc satisfaction questionnaire and perceived effort. All participants completed the sessions without adverse effects (100%), without SSQ symptoms reported. Post-gaming SUS was >75% in both sessions (range 75–80%). Post-gaming GEQ scores were 3.3–4.0/4 in both sessions. Immersive virtual reality video gaming is feasible in patients with mild-moderate PD, with positive usability and patient satisfaction, and no adverse effects.

## 1. Introduction

Parkinson´s disease (PD) is the most frequent neurological affectation in the elderly, along with dementia [1]. Prevalence is estimated between 0.3–1% in subjects older than 60 years, and 3% in people over 80 years [2]. According to the statistics up to 2016, 6.1 million patients suffer from PD globally [3]. Symptoms are divided into motor symptoms (bradykinesia, rigidity, resting tremor, and postural instability) and nonmotor symptoms (cognitive impairment, psychiatric symptoms, fatigue, pain or sleep disorders) [4].

Exercise therapies might promote improvements in the motor symptoms, [5] but also have an important role in the management of nonmotor symptoms [6], therefore, they are a first choice treatment option in PD and also in other Parkinsonian disorders [7].

Other highlights in the field in the approach to PD is the use of active video games (exergaming) as a rehabilitation tool. In fact, this is well documented. There is scientific evidence supporting the participation in exergaming programs designed for entertainment platforms such as Nintendo Wii, Microsoft Xbox or Sony PlayStation, which represents an economical and motivating alternative [8]. These programs have been applied to different groups: healthy elderly people [9], post stroke [10] and even Parkinson’s disease (PD) [11], and have demonstrated benefits in both physical and psychological aspects, improving physical activity levels, the performance of daily activities, balance, and cognitive functions in older people [12].

However, researchers have also reported limitations associated with the interface of the video games used and structural problems when applying them to more fragile groups [13]. Counteracting these limitations could make virtual reality (VR) a suitable tool for the application of therapeutic and rehabilitation programs, due to its adaptability to the patient, specificity to the pathology and expected high adherence [14]. 

Therefore, rehabilitation activities that are more engaging can be more effective compared to conventional rehabilitation [15,16].

The VR environment can be non-immersive, semi-immersive and fully immersive [17], and the presence and immersion of the system is increasing from the use of a PC (non-immersive), large monitors (semi-immersive) or CAVE rooms or head-mounted displays (HMD) (fully immersive) [18]. Gaming augmented with visual and audio feedback exploits neurophysiological reward mechanisms e.g., by engaging dopaminergic reward systems, which can enhance brain plasticity or could be beneficial in Parkinsonian disorders [19].

One such fragile group is Parkinsonians, who could benefit from working with Immersive virtual reality (IVR). However, few studies have addressed the implementation of therapeutic and rehabilitation programs in fully immersive settings in the general population and even fewer in Parkinsonians [20].

Therefore, the aim of this study was to describe the cases of PD patients who tried video games with a commercial HMD in a fully immersive environment and investigate its possible use as a therapeutic exercise tool in this population. Safety, usability, personal experiences, user satisfaction and effort post exergame were evaluated. 

## 2. Materials and Methods

Four participants diagnosed with PD (Table 1), with a Hoehn and Yahr stage II evolution of the disease, participated in the study. The Vigo Association of Parkinson (Vigo, Spain) was contacted by telephone to explain the project and they were invited to participate in the IVR sessions on a voluntary basis. Subjects’ exclusion criteria were inability to correctly respond to the assessment protocol according to the clinician´s judgment; presence of cardiovascular, pulmonary or musculoskeletal condition that according to physiotherapist judgment affects patients’ ability to participate in the study; presence of severe visual loss that could interfere with the ability to see the IVR simulation as well as vertigo, epilepsy and psychosis. All participants that fulfil selection criteria were invited to participate. Information was provided to the patients on objectives, duration, procedures, and voluntariness and informed consent was obtained.

**Figure 1:**
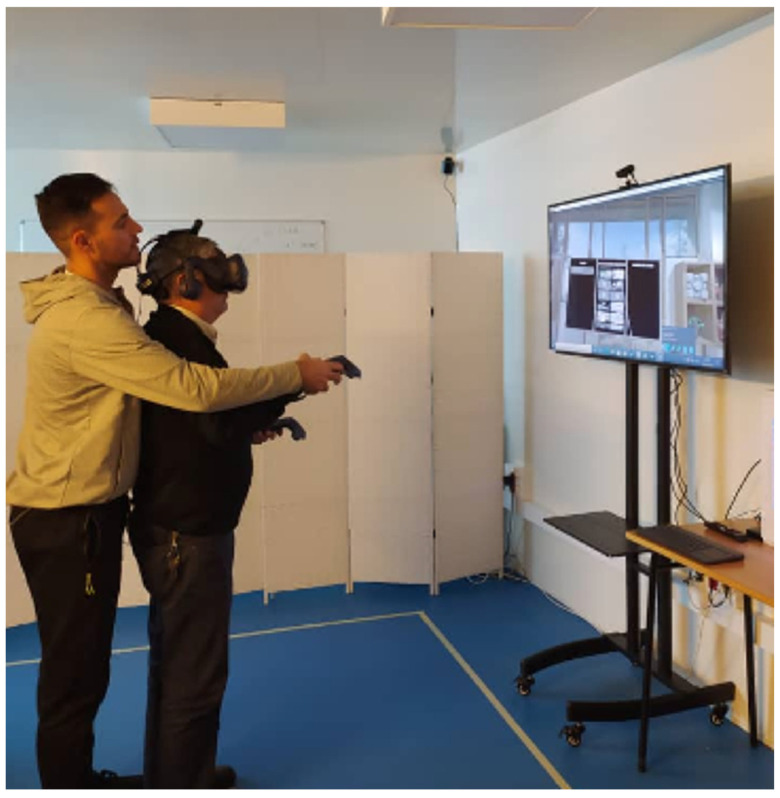
Participant during an individual session guided and supervised by a physiotherapist.

The immersive virtual environment was created using the HTC Vive ProTM commercial entertainment device. This system consists of a HMD, two handheld controllers, two external sensors to delimit the gaming surface, a wireless adapter and the Viveport software support (https://viveport.com accessed on 10 July 2021), supported by a desktop computer (CPU: Intel Core I7 7700 at 3.6 GHz, 1 Tb HDD Sata 3.5 and NVIDIA GeForce RTX 2070 GPUs). LED display was used to guide activities and set up technical aspects of the device. A 5 m^2^ play area was defined following the manufacturer’s installation recommendations and taking into consideration the dimensions of the area selected for the study. Two interventions were set on two different days and participants used IVR for 10–12 min approx., respectively. The researchers selected 1 experience (pre-training) and 1 game (training), planned as two parts of one session. Patients were informed of the need to end the session if they experienced discomfort or excessive fatigue. In view of the characteristics of the participants, the playing position chosen was standing. Each participant had an individual guided and supervised session by a physiotherapist with experience in PD (Figure 1).

**Figure 2:**
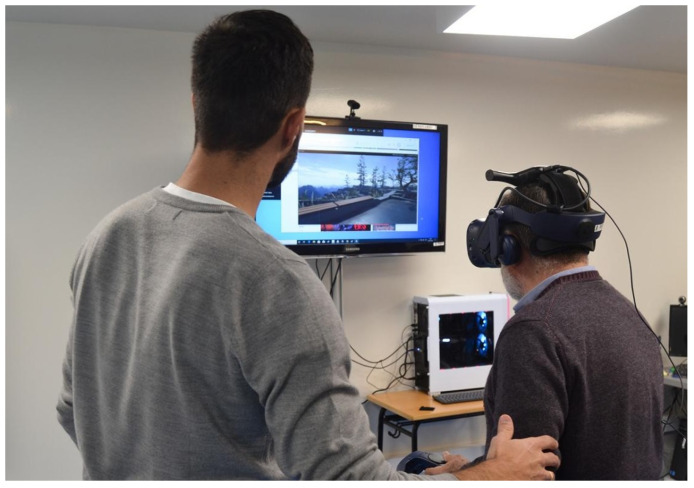
Participant during the introduction session (SteamVR Home) before the training session 1 placed in a mountain landscape.

Session 1: This was set up as an introduction to the IVR. It was composed of:

- An instructional talk and an explanation of how to handle the device, both by demonstration and by allowing the participants to try out the controls and the HMD.
- First part of the session: Introduction session (pretraining), the experience was Steam VR Home, which simulates being in a room with a door opening onto an exterior mountain landscape. Duration: 9 min approx. (Figure 2). The subjects moved around the environment, observing it and manipulating different objects. During this time, they answered questions that the therapists asked about the characteristics of the environment, in order to test their presence and immersion in the game, as well as performing body movements (real and virtual) and manipulating objects. This VR experience was chosen to provide a first contact with the IVR in a nice, fun and quiet virtual scenario to ensure good acceptability. At the end of the session, in a sitting position, the participants were asked if they had experienced discomfort linked to cybersickness.
- Then, in the second part of the session (training), the participants played the game BOX VR (available in the library of Viveport.com accessed on 10 July 2021), which simulates being in a gym. The participants must perform different boxing techniques (guard, jab, cross, hook, uppercut) and they must move their trunk, head, and lower limbs, to vary their position if required, as well as performing coordination movements. This game was chosen because it required considerable body movement (joint mobility, muscle power, muscle tone) and it provided different form of physical interaction, very similar to what is intended in a traditional physiotherapy session in PD. This part of the session took about 3 min.

**Figure 3:**
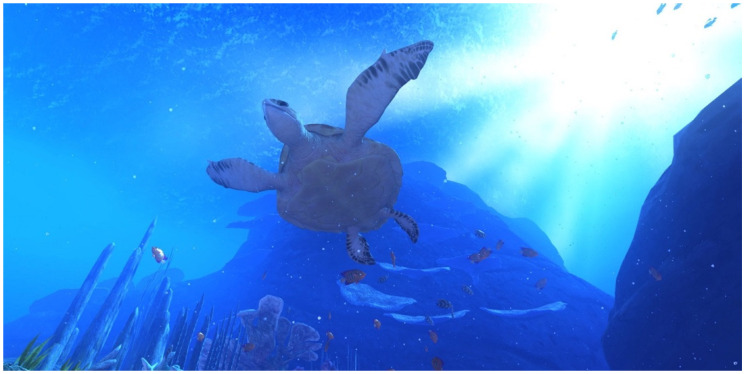
Screenshot of the acclimatization experience proposed (TheBlue) before session 2 placed at the sea.

**Figure 4:**
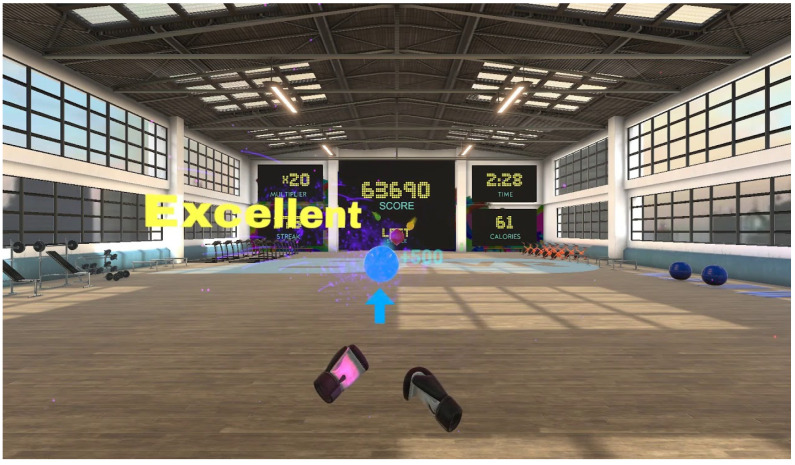
Screenshot of the exergame proposed (BOX VR) for training sessions.

Session 2: This session took place two weeks after session one. The acclimatization period was reduced to 5 min approx., using the TheBlue experience (pretraining), where the participants were located on the seabed (Figure 3). This experience can also be used for acclimatization to immersive environments, as, due to the environment and sound that accompanies it, it is very pleasant and relaxing. The participants moved around the seabed, observing it and interacting with objects using hands while walking. Again, at the end of the session, the participants were asked if they had experienced any discomfort linked to the presence of cybersickness. In the second part of the session (training), the BOX VR game was used again. This session was more demanding in terms of time and movements, incorporating squats and wide lateral movements of the trunk to avoid objects (balance training), and at the same time, to perform upper limb boxing movements to provide other PD physiotherapy modality—dual task training (Figure 4).

### Assessments

At the end of each session, safety of the immersive experience was evaluated using Simulator Sickness Questionnaire (SSQ) [21], usability of the system was evaluated using System Usability Scale (SUS) [22], and finally, personal experiences were collected using the Game Experience Questionnaire (GEQ-post game module) [23]. In order to reach an understanding about user satisfaction and perceived effort, an ad hoc satisfaction questionnaire was also applied to identify the strengths and weaknesses of the intervention and Borg’s perceived effort scale [24]. These assessment tools have been used in similar research [25,26,27] and are intended to assess the feasibility of using IVR exercise-based experiences. As control measures, during the exergaming task, the average heart rate was monitored by Mi Smart Band 4 wristband and Mi Fit 4.0.14 version app.

## 3. Results

All participants completed the training sessions successfully without any adverse effects in both sessions. Table 2 and Table 3 show the results of safety (cybersickness), usability, personal experiences (experience, tiredness and return to reality) and effort scores (perceived exertion and heart rate) for each session.

Satisfaction ad hoc questionnaire (Table 4) showed that all participants reported that it had been “a very good experience”; patient 3 indicated “it was a too short experience”. Further, the participants would repeat the experience; they thought that it would be useful for people with PD, and they would recommend it to other members of the Association.

## 4. Discussion

The goal of this study was to provide early insights into how feasible it would be to play with a commercial HMD in a fully immersive environment, for patients with mild-moderate PD, as Kim et al. [27] in 2017 did.

The results also showed that the IVR platform and the selected experiences lend validity to the proposition of developing an exergaming program as a rehabilitation tool for this population. Previous studies have reported that cybersickness was more likely in elderly people and Parkinsonians [27]. Our results, however, reinforce the safety of our IVR training, and the data collected by the SSQ did not reflect any symptoms. Although it is unusual not to observe sickness effects, this may be the result of the small number of IVR sessions or the sample size, an aspect that will be addressed in future studies. However, this seems to be a good starting point for approaching the treatment of Parkinson´s disease patients with IVR.

Satisfaction ad hoc questionnaire answers and SUS results offered valuable and positive information. GEQ-post game results support the proposed immersive environment by highlighting scores for positive experience items. Nevertheless, considering the group, we agree with other authors on the importance of maintaining guided and supervised sessions to prevent problems or dropping out of session [28]. 

Responding to the hypothesis of possible therapeutic approaches, both interventions were valid as a method of therapeutic exercise, which is so necessary in the Parkinsonian population. The choice of exergame was based on characteristics such as it is demanding a quick response to the presentation of stimuli and for the need to perform movements on different planes of the upper and lower limbs and trunk, aspects which require special focus in the PD population [29]. Besides, the exergaming proposed will achieve enough exertion to be considered exercise and it allows cardio-vascular work, similar to traditional recommended activities in PD management [5]. 

The virtual and real environment was considered safe and the activities appropriate, fun and motivating. Taking into account that in the first intervention, the perceived effort was considered low by all the participants, the second intervention was seen as more demanding. This increase in demand was a result of having to work and provide quick results to the presentation of the stimuli, an aspect that surely helps in the improvement of coordination, proprioception and treatment of bradykinesia, in addition to aspects of postural control and balance training.

We note that physical exertion was similar in both sessions, although perceived exertion was greater in the second session as we expected. However, heart rate measurements yielded disparate values in one case, where the subject’s average HR showed significantly higher values in the second session. This fact supports the idea that the intensity of work undertaken in the second intervention could be established as a reference session for a future longer-term training, although we suggest that a future research direction is to provide standardized exercise protocol in people with PD if the aims are, for example, to improve gait, balance or strength. For this, it would be advisable to incorporate other assessment tools more specific to PD such as PDQ-39 or MDS-UPDRS, as well as assessment gait and balance scales such as TUG or MiniBest test.

### Limitations

These preliminary outcomes are promising, but there are some limitations to our study. One of them is that most of the variables are assessed by questionnaire-based methods. The positive predisposition of the sample may have favored the findings. In addition, the good results may also be supported by the low number of participants, as well as by the fact that the study was designed as a one-time experience. Future research with representative samples should include objective tests in addition to questionnaires, treatment protocols of several sessions per week, for several weeks, and patients with different PD conditions. Furthermore, the experiences were only evaluated after two interventions and with a similar virtual scenario profile. Current investigations are underway in our lab to examine different conditions: more training sessions, foot trackers, alternative games’ levels or patients with advanced disease.

## 5. Conclusions

In this study, we informed our findings about this emerging virtual exercise tool. In the cases reported, PD was not a limitation and the sessions are safe. Immersive virtual reality video gaming was feasible in the patients with mild-moderate PD presented, with positive experience opinions, good usability as well as positive patient satisfaction and no adverse effects.

There is commercial software, HMDs and IVR exergames that can provide enough exertion to be compared to traditional exercise activities such as walking or dancing (highly recommended in PD physiotherapy clinical guidelines).

Future research could involve clinical trials, with long-term training effects and a larger sample size, encouraging the evaluation of physical, psychological and social variables in the Parkinsonian population, or even their own aspects to this disorder such as improving balance, strength, gait or functional independence.

## Figures and Tables

**Table 1:** Demographic characteristics of the participants.

	Patient 1	Patient 2	Patient 3	Patient 4
Age (years)	64	61	53	71
Gender	Male	Male	Female	Male
BMI (kg/m^2^)	29.40	29.40	33.32	24.05
Time since PD diagnosis (years)	5	2	2	13

BMI: Body Mass Index; PD: Parkinson’s disease.

**Table 2:** Results of IVR training sessions.

	Patient 1	Patient 2	Patient 3	Patient 4
Session 1 (training)				
SSQ	No Symp	No Symp	No Symp	No Symp
SUS	75/100	75/100	100/100	80/100
GEQ-post game				
*Positive Experience	3.3/4	2.5/4	4/4	3/4
*Negative Experience	0.16/4	0.33/4	0/4	0.33/4
*Tiredness	0/4	0/4	0/4	0/4
*Return to Reality	0/4	0.66/4	1.66/4	0.33/4
Session 2 (training)				
SSQ	No Symp	No Symp	No Symp	No Symp
SUS	87.5/100	90/100	100/100	90/100
GEQ-post game				
*Positive Experience	3.3/4	2.5/4	3.66/4	2.83/4
*Negative Experience	0.16/4	0.16/4	0/4	0.16/4
*Tiredness	0/4	2/4	2/4	0.5/4
*Return to Reality	0.33/4	1/4	1.3/4	0.33/4

Symp: Symptoms; SSQ: Simulator Sickness Questionnaire; SUS: System Usability Scale; GEQ: Game Experience Questionnaire.

**Table 3:** Effort scores (perceived exertion and heart rate) for each session.

	Patient 1	Patient 2	Patient 3	Patient 4
Session 1 (training)				
Total time	11:25	12:07	11:40	11:20
Time (TS)	2:37	2:37	2:37	2:37
Borg score (TS)	3/10	2–3/10	1/10	2–3/10
HR mean -b/m- (TS)	94	100	100	100
Session 2 (training)				
Total time	11:03	10:46	11:22	11:48
Time (TS)	5:53	5:53	5:53	5:53
Borg score (TS)	5/10	7/10	8/10	7/10
HR mean -b/m- (TS)	86	90	103	122

b/m: beats/minute; HR: Heart Rate; TS: Training Session.

**Table 4:** Satisfaction ad hoc questionnaire.

Questions	Answers, n (%)
1. How was the experience? (A) What did you like the best? (B) Was there anything you didn´t like?	“Good and/or/very good” (100%) “How entertaining and fun the experience was” “How real things seemed” “The game” “Being under water” “No” (75%) “Too short”
2. Would you repeat the experience with VRI?	“Yes” (100%)
3. Would you recommend the experience of VRI?	Yes” (100%)
4. Do you think the exercise is suitable for people of your age? Why?	“Yes” (100%) “You can experience new sensations” “It motivates me to do exercise” “It helps you stay in shape and it´s relaxing” “It´s doing exercise in a fun and different way”
5. Free comments	“The best experience I´ve had to date” “I am feeling so good”

## Data Availability

The data presented in this study are available on request from the corresponding author.
